# The Quality of Emulsions with New Synthetized Lipids Stabilized by Xanthan Gum

**DOI:** 10.3390/biom11020213

**Published:** 2021-02-03

**Authors:** Małgorzata Kowalska, Paweł Turek, Anna Żbikowska, Monika Babut, Jerzy Szakiel

**Affiliations:** 1 Department of Management and Product Quality, Faculty of Chemical Engineering and Commodity Science, Kazimierz Pulaski University of Technology and Humanities, Chrobrego St. 27, 26-600 Radom, Poland; m.babut@uthrad.pl; 2 Department of Non-Food Product Quality and Safety, Cracow University of Economics, Rakowicka St. 27, 31-510 Cracow, Poland; turekp@uek.krakow.pl (P.T.); szakielj@uek.krakow.pl (J.S.); 3 Faculty of Food Assessment and Technology, Institute of Food Sciences, Warsaw University of Life Sciences (WULS-SGGW), Nowoursynowska St. 159c, 02-776 Warsaw, Poland; anna_zbikowska@edu.sggw.pl

**Keywords:** lipids, enzymatic modification, Turbiscan test, emulsion, xanthan gum

## Abstract

The study investigated the quality of emulsions containing rabbit fat modified with vegetable oil. The modification of the fat and introducing it as a fatty base into the emulsion was dictated by consumer preferences. Emulsion systems containing various fatty bases and viscosity modifier contents were evaluated in the terms of their stability (by means of Turbiscan test), texture properties, color, and viscosity. Moreover, the emulsions were assessed by a sensory panel in the context of the intensity of the following parameters: color, fragrance, consistency, greasiness, and hydration. The same characteristics were also subject to consumer evaluation. The results of the sensory assessment showed the sensory panel attributed higher scores to consistency and skin hydration to the emulsions formed with modified fats; these systems were more appreciated by consumers as well. The results confirmed a major role of sensory determinations in the development of new emulsion products. They also provide knowledge on modifications to product characteristics that would lead to the best possible quality and consumer acceptance. This research has also reaffirmed that looking for new fats among waste fats is becoming a solution to finding new fatty bases for emulsions. The natural origin of these components, and thus their agreeability with the human body, appear noteworthy as well. Enrichment with unsaturated fatty acids is an added advantage of the enzymatic modification of rabbit fat with pumpkin seed oil and can be applied not only for food but also for skin applications.

## 1. Introduction

Natural products, including cosmetics preparations, are becoming more and more popular, and their health benefits are widely recognized [1]. Nowadays, with sustainability becoming an important issue, consumers more often are looking for natural, ecological products. The increased interest in such products results from the care for both the environment and health [2]. In addition, products with a reduced amount of irritating ingredients and an increased amount of natural ingredients are becoming a more and more sought after by consumers. This is confirmed by cosmetics market analysis that an increasing percentage of consumers buy natural cosmetics [3]. The market forecasts assume that global sales of organic cosmetics will increase from $34.5 billion (2018) to $54.5 billion (2027) [3].

Generally, emulsions have become a universal form of many products that, owing to carefully selected ingredients, are characterized by appropriate physical, chemical, and functional properties [4]. The knowledge of the physical chemistry of dispersion systems, namely, their texture, viscosity or stability, provides necessary information for designing an emulsion system with desirable quality characteristics [5]. From the thermodynamic point of view, emulsions are systems of a low kinetic stability, therefore, stabilizers, i.e., emulsifiers, maturing inhibitors, and texture modifiers are added to enhance their shelf-life and stability and to improve the system overall.

Since consumers increasingly demand ‘label-friendly products’ based on natural and sustainable ingredients, manufacturers are interested in replacing synthetic surfactants with natural emulsifiers [6]. Biopolymer-based emulsifiers are commonly used to stabilize emulsion systems naturally [7]. Xanthan gum, a micromolecular, hydrophile biopolymer is produced through carbohydrates fermentation by *Xanthomonas campestris* bacteria [8] and is the most frequent used biopolymer-based emulsifier. This hydrocolloid can enhance a system stability by raising apparent viscosity of a dispersing phase even at relatively low concentrations [9]. Specific rheological properties (pseudoplasticity) of xanthan gum aqueous solutions contribute to the sensory properties (flavor sensations) of a final product that are acceptable by consumers [10].

Aside from additives to emulsion systems, a fatty phase type or, to be more precise, type of fat introduced to a system, is an important factor determining both physicochemical and rheological properties. Physicochemical properties of fat blends that provide bases for emulsion systems have a decisive influence on functional attributes of the final products [11].

The enzymatic modification of fats is performed to produce lipids of novel structures, adequate plasticity, and melting point. The interesterification of hard fats with vegetable oils allows for obtaining new triacylglicerols with increased nutritional value, especially when it comes to ingredients such as essential unsaturated fatty acids [12].

Fat microstructure changes in the process of interesterification, resulting in the formation of smaller crystals with regular dimensions, which assures a homogeneous consistency without grainy effects [13]. These aspects have a decisive effect on such sensations as the mouth feel and spreadability, and thereby an overall sensory assessment of fat products. 

According to the information provided by Rojas et al. [14], pumpkin seed oil is an oil containing about 80% of unsaturated fatty acids (mainly oleic and linoleic acids). The oil also contains phytosterols, proteins, antioxidants, and vitamins [15].

One of the increasingly attractive hard fats is rabbit fat. The composition of rabbit meat lipids is beneficial due to the high content of monounsaturated and polyunsaturated fatty acids (FA), as well as of valuable odd-numbered straight-chain and methyl-branched-chain fatty acids [16,17]. An appropriate proportion between PUFAn-6 and PUFAn-3 existing in rabbit meat and fat can be responsible for the prevention of some correlated diseases, such as hypercholesterolemia-related heart attack and strokes [18]. In addition, the presence of acids like CLA, docosahexaenoic, and eicosapentaenoic acids, confirms that this fat is healthy and safe for human beings [19].

Taking into account the beneficial characteristics of both rabbit fat and pumpkin oil they seem to be desired ingredients in food, cosmetic, or even medicinal formulations.

The aim of the study was to increase the nutritional value and modification of rabbit fat with pumpkin seed oil. The newly produced fats were used as the fatty bases of model emulsions. Such products based on natural components can be used not only in the food industry, but also as medicinal or cosmetic preparations.

## 2. Materials and Methods 

### 2.1. Materials

The following ingredients were used in experiments: cold pressed pumpkin seed oil (PSO) was purchased at a local market (Oleofarm s.c., Wrocław, Poland); rabbit fat (RF) was obtained from a private farm. Lipase (sn-1,3 regioselective) immobilized on Immobead 150 from *Rhizomucor miehei*, ≥300 U/g (Sigma Aldrich) was used as a catalyst. Xanthan Gum (Brenntag, Poland) was used as a thickener for emulsions. Sodium benzoate was applied as a preservative (Orff Food Eastern Europe). 

### 2.2. Methods

In order to obtain a fatty base, rabbit fat and pumpkin seed oil blends were prepared with the following weight ratios: 3:1; 3:3; 1:3. Each blend was divided in two parts, and one part was subjected to an enzymatic process. 

#### 2.2.1. Enzymatic Modification (EIE) Reaction 

The prepared blends were transferred into three Erlenmayer flasks (250 mL). Then, the flasks containing the prepared fat blends were thermostated at the reaction temperature of 60 °C ± 0.5 °C in a shaker equipped with a water bath (SWB 22N, Labo Play, Poland) for 15 min. After this time, 5% (relative to the fat blend mass) of an sn-1,3 regioselective lipase and 1.1% (in relation to the fat blend mass) of distilled water were introduced into the flasks. The introduction of water was aimed at shifting the equilibrium of the interesterification reaction towards hydrolysis to produce more mono- and diacylglycerols, which act as emulsifiers in the dispersion systems produced later. The flasks with the prepared blends were shaken vigorously for 6 h. After this time, the process was stopped by hot filtration in order to separate the enzyme from the modified blends on a Büchner funnel with a paper filter.

#### 2.2.2. Preparation of Emulsions

Twelve dispersion systems with different fatty bases and viscosity modifier contents were prepared (Table 1). As mentioned earlier, the fat phase of the emulsions consisted of modified (E1–E6) and unmodified (E7–E12) fat blends. Aqueous phases were obtained by dispersing of an appropriate amount of hydrocolloid (xanthan gum) in distilled water for 1 min (Table 1). Both the phases (oil and water) were heated to 55–60 °C and then homogenized for 4 min using a mechanical homogenizer equipped with an S18G–19G dispersing head (T18 digital ULTRA-TURRAX, IKA, at a constant speed of 18,000 rpm). The emulsions were cooled to room temperature and 0.3% preservative (sodium benzoate) was added. Each emulsion was prepared as a 100 g product.

#### 2.2.3. Texture Analysis

The texture assessment was performed using a Texture Analyzer CT3 (Brookfield Engineering Laboratories, Inc., Middleboro, MA, USA) equipped with Brookfield Texture Pro CT software. The tests were carried out using a one-cycle compression and nylon spherical shaped probe (TA43; 30 mm internal diameter; 50 mm depth). The test and return speed was 0.5 mm/s, pretest speed 2 mm/s, with a target depth of 10 mm, the trigger load was 1 g, and the data rate was 10 points/s. The instrumental textural characterization of the emulsions employed hardness and adhesiveness. Freshly prepared emulsions and emulsions after 30 days of storage at a room temperature were tested. The emulsion samples were subjected to texture profile analysis (TPA) with three replicates.

#### 2.2.4. Dynamic Viscosity

The dynamic viscosity of the emulsions was evaluated using a rheometer (DV-III Ultra Brookfield Engineering Laboratories, USA), the model including a helipath spindle set, using T-bar spindle No. 93 (C) at 20 rpm. The results of the study were showed as a diagram illustrating mean values obtained from three measurements. The measurements were performed 24 h and 30 days after the preparation. 

#### 2.2.5. Instrumental Determination of Emulsion Color 

The measurements of the dispersion systems color were carried out in a CIE L* a* b* system [20] by means of the reflection method using a colorimeter (model CR 400, Konica Minolta, Japan). The colorimeter was equipped with a receptor head with the diameter of 8 mm and a xenon lamp (the source of light). The measurement time was 1 s. The preparations for the measurement began with calibration on a white calibration plate with the following parameters: L = 87.5; a = 0.3155; b = 0.3227. The color of the emulsions was determined on freshly prepared and stored emulsions with three replicates, then the values were averaged. Moreover, the overall difference in color (∆E) and the color saturation parameter C* were calculated to describe the color changes caused by storage conditions. The following formulas were used for the calculations:

(1) $$C^{*}=\;\sqrt{{\left(a^{*}\right)}^{2}+{\left(b^{*}\right)}^{2}}\;$$

(2) $$∆E=\;\sqrt{{\left(L_{0}^{*}-L^{*}\right)}^{2}+{\left(a_{0}^{*}-a^{*}\right)}^{2}+{\left(b_{0}^{*}-b^{*}\right)}^{2}}\;$$

where:

∆E—color difference between freshly prepared and stored emulsions,
C*—difference in color saturation,
L*—intensity of color brightness from 0.00 (black) to 100.0 (white),
a*—indication of a value between red and green,
b*—indication of a value between yellow and blue [21].

#### 2.2.6. Stability Evaluation

The kinetic stability of the prepared emulsions was evaluated by multiple light scattering using a Turbiscan Lab Expert Stability Analyzer (Formulaction, L’Union, France). The light source was an electroluminescent diode emitting near-IR light (λ = 880 nm). The backscattering detector collected the light backscattered by the emulsions at an angle of 45° with respect to the source, while a transmittance detector received the light that passed through the samples at an angle of 180° [22]. 20 mL O/W emulsion samples were placed into cylindrical measuring cells and analyzed by the Turbiscan Lab Expert. Two synchronized optical sensors (receiving transmitted (T) and backscattered (BS) light) scanned the entire height of the emulsions (42 mm) at 25 °C for 20 s. The samples were stored at a room temperature and determinations were carried out for 30 days, every 3–4 days. 

The stability of the emulsions was presented as a backscattering profile (∆BS) and Turbiscan Stability Index (TSI). Both the parameters were calculated according to the following formulas:

(3) $$BS\;\approx \;1/\sqrt{\lambda^{*}}$$

(4) $$\lambda^{*}\;\left(\phi ,d\right)=\;\frac{2d}{3\phi \left(1-g\right)Q_{s}}$$

(5) $$TSI=\;\sqrt{\frac{\Sigma_{i=1\;}^{n}{\left(X_{i}-\;X_{BS}\right)}^{2}}{n-1}}$$

where:

λ*—is the photon transport mean free path in the analyzed dispersion,
ϕ—is the volume fraction of emulsion particles,
d—is the mean diameter of particles,
g, Qs—are the optical parameters given by the Mie theory,
X_i_—is the average backscattering for each minute of measurement,
X_BS_—is the average X_i_
n—is the number of scans [22].

#### 2.2.7. Sensory Analysis

The sensory team was qualified and trained using specialistic literature [23] and relevant standards [24,25,26,27,28,29]. Sensory sensitivity was tested for sight, smell, and touch. The initial training stressed the ability to identify particular stimuli and order samples with varying intensity of tested characteristics. The evaluation was conducted by 12 assessors (As per ISO 5492:2008 Sensory analysis. Vocabulary—selected assessors are selected for their ability to carry out sensory testing.). Only females were the assessors in this test. The samples were designated with three-digit codes; however, the results were presented as symbols used in discussions of instrumental testing for the sake of text legibility. Sensory profiles of the test samples were developed to represent intensities of individual characteristics, namely, color, fragrance, consistency, greasiness, and hydration. The intensities of selected factors were evaluated by means of a 100 mm graphic scale provided with boundary definitions appropriate to each factor. 

The samples were assessed at room temperature. The resultant sensory profiles were compared with consumer assessments in order to indicate the characteristics differentiating the samples and potentially affecting the palatability results of the consumer testing. The testing by the sensory analysis team was conducted on the 30th day of the product storage.

#### 2.2.8. Consumer Testing

The consumer testing relied on the methods of consumer determinations in controlled conditions as laid down in ISO 11136:2014. Each product was assessed by a minimum of 60 females aged 20-30. A 9-point hedonic verbal scale was applied, with 1 meaning ‘dislike extremely’ and 9 ‘like extremely’ [23]. Due to the high number of samples, each consumer evaluated a maximum of 6, with the consumers being presented with varied sample arrangements. A total of 69 assessments were obtained for each sample. The consumer testing was carried out between the 30th and 32nd day of storage.

To verify similarity or variation across the objects tested in respect of the selected set of characteristics (color, fragrance, consistency, greasiness, and hydration), PROFIT (PROperty FITting) analysis was applied to the consumer assessments. The method combined results of multidimensional scaling and multiple regression analysis [30]. Similarities between the tested samples were computed based on the inputs. A matrix of Euclidean distances was calculated on the basis of all the averaged assessments of the test samples. Quality of matching of reproduced and input data was measured by means of STRESS (Standardized Residual Sum of Squares) 

## 3. Results and Discussion 

### 3.1. Texture Analysis

**Figure 1:**
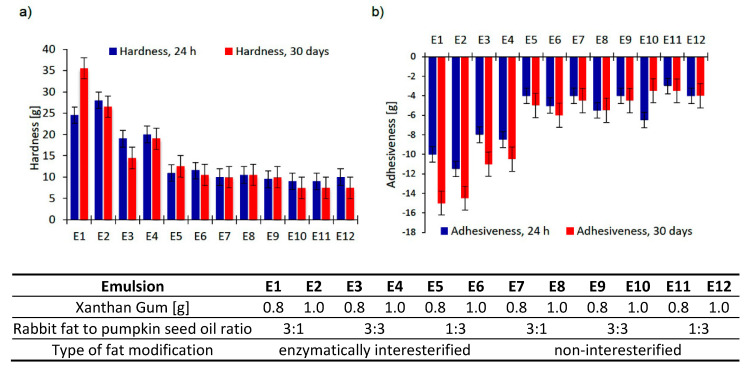
Texture parameters ((**a**) hardness, (**b**) adhesiveness) for emulsions after 24 h and 30 days from their preparation (as a mean value of three determinations ± SD).

Texture measurement is an instrumental method of measuring mechanical product properties (related to macroscopic, microscopic or molecular structure) that, as they are closely correlated with sensory testing (conducted by sight, hearing, touch, and kinaesthetics), are mainly applied to cosmetic and food products [31]. Texture characteristics are measured by compressing a sample with a probe twice (2 cycles). The parameter of hardness represents a force necessary to compress a sample, while adhesiveness denotes work needed to overcome the strength of a probe adhering to the surface of a system analyzed [32]. Texture properties were analyzed for freshly prepared emulsions and after 30 days of the emulsion storage at room temperature. The results are shown in Figure 1, considering hardness (Figure 1a) and adhesiveness (Figure 1b) of the dispersion systems tested.

Emulsions based on interesterified (E1–E6) fat blends were characterized by greater hardness values than the emulsions based on non-interesterified (E7–E8) fat blends; this was particularly true of the first four emulsions E1–E4. For the majority of emulsions where the fatty base was an interesterified fat, a decrease in hardness after a 30-day storage period was observed. Only for the emulsions E1 and E5, this parameter found to rise to 11 g and 1.5 g for E1 and E5, respectively. Taking into account the type of fat in these emulsions, no relationship was found between the amount of rabbit fat and the addition of oil. Therefore, it can be supposed that variations of this parameter could have been driven by the process of interesterification itself and by fatty acids positioned in triacylglycerol molecules, which finally changed system consistency as a result of emulsion formation and viscosity modifier additions. Among the freshly prepared emulsions, the maximum hardness (28 g and 24.5 g, respectively) was noted for E2 and E1 emulsions, containing the highest content of enzymatically modified rabbit fat, whereas E10 and E11, i.e., emulsions including physically modified fat, displayed minimum hardness (9 g and 10 g, respectively). In general, the emulsions containing mixed fat showed no substantial variations of this parameter (E7, E8, E9) or such variations were negligible (E10, E11, E12), regardless of the amount of xanthan gum introduced to a system.

Adhesiveness is another parameter having a decisive effect on texture characteristics of a product. Maximum adhesiveness was noted for the emulsions E2 and E1 (−11.5 g and −10 g, respectively) 24 h after their preparation (Figure 1b). The results correspond to the maximum hardness values (Figure 1a) for these samples. The results of the texture properties showed that the greater the content of pumpkin seed oil, the lower both the hardness and adhesiveness in respect of the emulsions based on structured lipids.

With regard to the quantities of the viscosity modifier introduced to the emulsion systems containing enzymatically modified fats, the emulsions containing 1% of hydrocolloid were found to exhibit higher hardness and adhesiveness 24 h after preparation than the emulsions including 0.8% of this compound. A similar tendency was observed in the case of adhesiveness of the emulsions based on mixed fats. Neither hardness nor adhesiveness displayed any tendencies after the period of storage.

### 3.2. Dynamic Viscosity 

Viscosity is the parameter largely responsible for stability and shelf-life of dispersion systems during storage [33]. Changes of emulsion viscosity at the time of storage are inevitable, yet undesirable to both consumers and manufacturers. To extend the stability of consistency and thus viscosity of dispersion systems, production engineers introduce stabilizers, thereby modifying rheological properties of dispersion systems [4]. 

**Figure 2:**
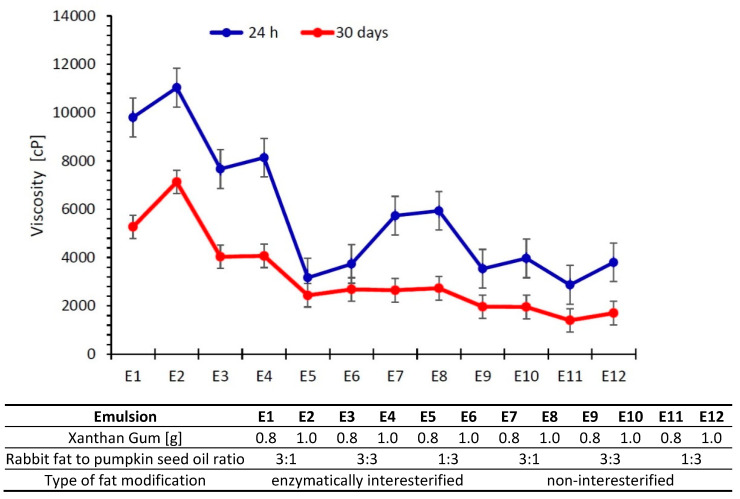
Viscosity of emulsions after 24 h and 30 days from their preparation (as a mean value of three determinations ± SD).

Emulsion E2 displayed the greatest viscosity after preparation as well as storage (11030 cP—24 h after preparation and 7127cP—after 30 days) (Figure 2). The lowest values of this parameter were noted for E5 and E11, i.e., systems containing a higher pumpkin seed oil content and with 0.8% of xanthan gum. Regardless of whether a fat was modified, the greater the content of pumpkin seed oil in the fatty phase of the dispersion system, the lower the viscosity. The results are in agreement with those obtained by [34] for emulsions based on enzymatically modified blends of rabbit fat and pumpkin seed oil including carboxymethylcellulose as a viscosity modifier.

After 30 days of storage, all the emulsions exhibited lower viscosity values (Figure 2). Papalamprou et al. [35] also observed that apparent viscosity of oil-in-water emulsions containing 0.1 or 0.25% of xanthan gum declined during storage, which they attributed to flocculation and coalescence of the dispersed phase droplets. Declining viscosity in storage was also noted by Saharudin et al. [36], who studied emulsions containing between 0.2 g to 1.0 g of xanthan gum.

The emulsions including 1.0% of xanthan gum were found to exhibit greater viscosity than those containing 0.8%, regardless of the time of storage or whether an emulsion contained interesterified or mixed fat.

### 3.3. Instrumental Determination of Emulsion Color 

**Figure 3:**
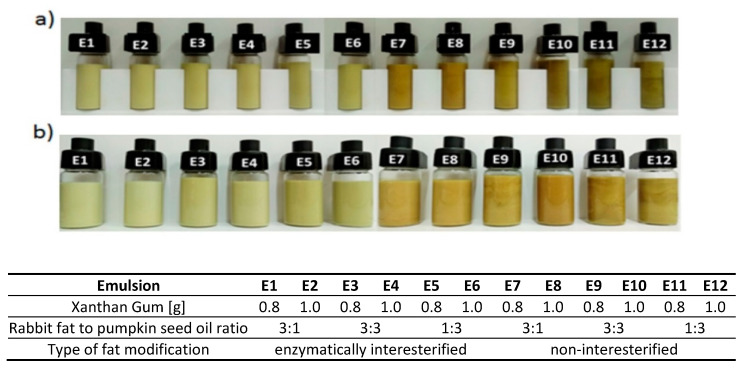
The appearance of emulsions E1-E12 (**a**) after 24 h and (**b**) 30 days from their preparation.

The color of an emulsion depends on system droplet interactions with light waves and the associated effects of reflection, adsorption, transmission, and scattering [37]. The results of color evaluation, i.e., L*, a*, b*, C* values and color difference ∆E were presented in Table 2. The results showed that values of lightness (L*) were greater for the emulsions based on structured lipids, which evidenced of their brighter color (Table 2, Figure 3). The values of this parameter decreased after 30 days of storage of all the emulsions by approx. 3.7, proof of the system darkening.

All the freshly prepared emulsions exhibited positive a* values, evidence of red tones in the systems. After 30 days of storage, the parameter became negative for all the emulsions, proof of the green tones. Such observations confirmed the dynamic variability of the systems over time and thus provided information about their quality. Values of b* increased by an average of 2.1 in the case of E1–E6 and approx. of 1.3 for E7–E12 after 30 days of the storage compared to its initial values. The growing intensity of yellow after the time of storage is evidence of changes in the emulsions as well. However, a more precise definition, i.e., the type of changes affecting the quality, was indicated by the analysis of the backscattered light intensity and the analysis of the TSI. The evaluation of the three color parameters (L*, a*, b*) was presented as total color difference (∆E) that addresses color differences between freshly prepared and stored emulsions. Changes of the parameter were minimum for E2 and E1 (1.9 and 2.7, respectively) and maximum for E9—4.5. Chudy et al. [38] interpret ∆E values between 3.5 and 5 as significant changes during the time of storage, while its values in the range 1 < ΔE < 2 as small differences that are perceptible only to those experienced in noting nuanced colors. Accordingly, changes of system color were significant for all the emulsions except for E2. Jochen and McClements [39] and Chantrapornchai et al. [40] believe color changes in emulsion systems arise from changes of droplet sizes at the time of storage, namely, L* rises as droplet concentration increases and their sizes decrease.

A visual assessment of freshly prepared emulsions indicated the emulsions based on enzymatically modified and physically mixed fats were brighter than the same emulsions after 30 days of storage (Figure 3). On the other hand, freshly prepared emulsions containing enzymatically modified fats exhibited brighter and more homogeneous color distribution compared to the same emulsions containing unmodified fats. This means the modification process contributed to a greater color homogenization of the emulsions based on these fats. The emulsions with maximum oil content, both as part of interesterified and mixed fat blends providing fatty system bases, displayed the most intensely dark shades. No effects of xanthan gum addition on color intensity or homogeneity could be observed.

### 3.4. Stability Evaluation

Colloidal and dispersion systems are unstable by nature, although they may be considered stable if the degree of destabilization is sufficiently low during the anticipated time of product storage. The analysis of the destabilization processes taking place is of great importance during the qualitative assessment of a system [41]. The selection of an appropriate method of identifying the phenomenon that occurs can be difficult as systems are frequently strongly concentrated and opaque [42]. According to Lemarchand et al. [43] an accurate method that allows to detect physical destabilization processes related to the migration of particles (sedimentation, creaming) or a change in their size (flocculation, coalescence) is the Turbiscan test, i.e., an analysis based on the evaluation of the transmission or backscattering light intensity directed to the sample.

**Figure 4:**
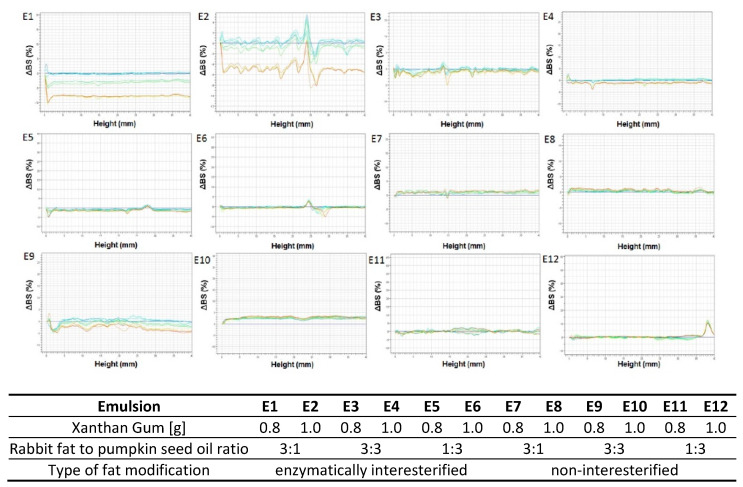
Delta backscattering profiles (ΔBS) as a function of the sample cell height (mm) for emulsions E1–E12 stored 30 days. Lines correspond to subsequent measurements performed in 3–4 days intervals, where blue line is Day-0 and red line is Day-30.

An analysis of the profiles found maximum ∆BS variations for E1 and E2, i.e., more than 8% and 4.5%, respectively, after 20 days of storage (Figure 4). The changes resulted from increasing average particle sizes (non-overlapping curves in the medium part of the diagram) and were evidence of such destabilization processes as flocculation or coalescence [44]. The effect was noted for the emulsions E3 and E9 as well, though to a far smaller extent (Figure 3). Destabilizing changes were not visible for the remaining emulsions based on interesterified (E3–E6) and non-interesterified fats (E7–E12).

Considering the variable xanthan gum contents of emulsions with the same fatty base, insignificant particle size variations were observed. The curve overlapping was more marked for the emulsions with greater amount of xanthan gum (1.0%) – except for E2. Gao et al. [45], Hanazawa and Murray [10], and Raymundo et al. [46] claimed that polysaccharides like xanthan gum, owing to gel-producing properties that restrict droplet movements, enhance system stability, thereby preventing destabilization processes. Velez et al. [47] observed that > ~ 0.1% of a polysaccharide needs to be added to produce a stable dispersion system. Meanwhile, Traynor et al. [48] carried out studies of emulsions containing 0.01 to 3% *w*/*v* of xanthan gum and concluded 0.28% *w*/*v* is the optimum concentration providing for stability of emulsion systems.

Considering the above, 0.8% of xanthan gum produced a stable emulsion system. This is reaffirmed by Turbiscan test results for emulsions based on non-interesterified fats without addition of any emulsifiers. These emulsions were only stabilized with the hydrocolloid and the ΔBS profiles recorded by Turbiscan were not far different than the results for the emulsions containing interesterified fats with mono- and diacylglycerols. The authors are of the opinion that the presence of these compounds may be important to extend shelf-life, however, any such changes could not be noted as the experiment was completed.

**Figure 5:**
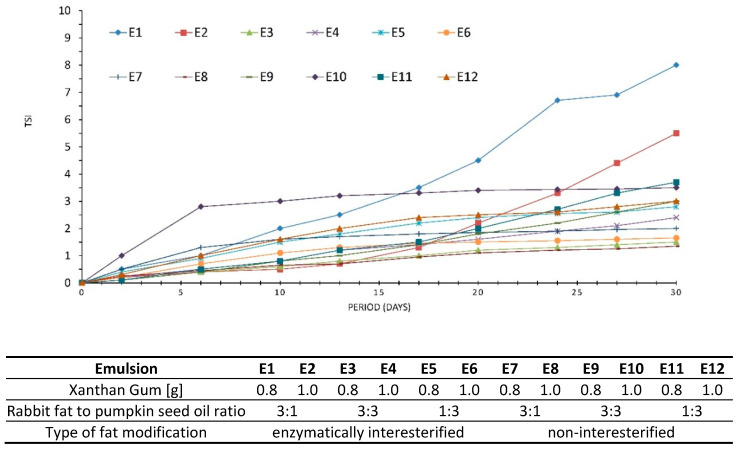
TSI values of emulsions E1–E12 during 30 days of storage. Each point corresponds to subsequent measurements performed in 3–4 days intervals.

Turbiscan Stability Index (TSI), otherwise known as the global destabilization parameter [42], is another parameter carrying information about system stability. TSI values range from 1 to 100, the higher values denoting presence of substantial destabilization processes in a system [49]. Obtained data suggest the emulsions E2 and E1 displayed the highest destabilization kinetics. Their TSI values were 5.5 for E2 and 8.0 for E1, respectively (Figure 5). Ren at al. [50] claim a system under analysis can be considered stable as a function of time if TSI is below 4. Therefore, E3–E12 were characterized by a relatively good stability during 30 days of storage, since their TSIs ranged between 1.35 and 3.7. No clear tendencies towards stability variations could be observed in respect of the hydrocolloid content. This can mean both 0.8 and 1.0% of the xanthan gum caused similar effects, therefore, its lower quantity seems more economically viable.

### 3.5. Sensory Analysis

**Figure 6:**
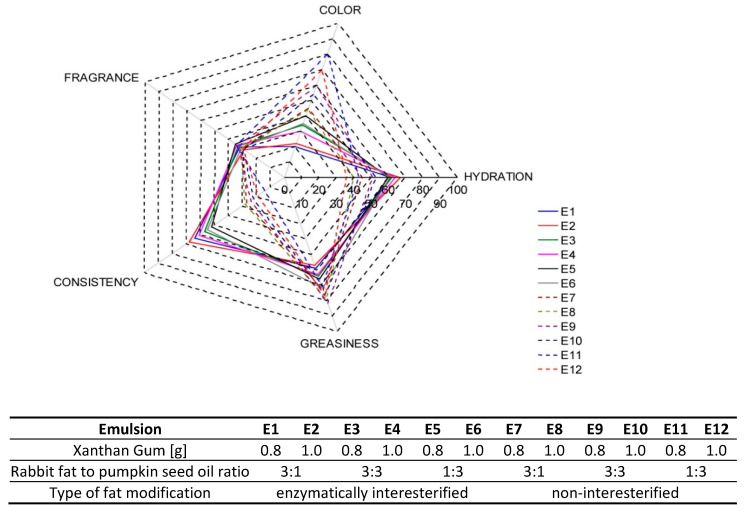
Sensory profile of emulsions E1–E12—evaluation of the sensory panel.

The sensory assessment performed by a team of 12 selected assessors pointed to maximum variations of perceived parameter intensity in respect of color and consistency. In the latter case, significant differences were noted between the emulsions containing and not containing interesterified fats (Figure 6).

In the assessors’ opinion, the emulsions E1–E6 were characterized by a more compact and homogeneous consistency, which complied with the greater hardness determined for the emulsions containing interesterified fats. As Moravkova and Filip [51] report, the results of instrumental analysis may serve as a good alternative method of determining basic sensory perceptions, which is affirmed in this study for the parameter assessments under discussion. A similar trend could be observed in the evaluation of hydration. The skin on which emulsions based on interesterified fats were applied was assessed as more hydrated than the skin on which the emulsions based on non-interesterified fats were applied. The sensory team noted a maximum hydration (67 units) after application of an emulsion based on modified fat where the ratio of animal to vegetable fat was 3:1 and 1.0% of the thickener had been added (E2). As far as non-interesterified samples are concerned, the emulsion E12, with a greater vegetable oil content (36 units), was rated the poorest. The evaluations varied the most in relation to color intensity of E1 = 20 and E11 = 80. The information from the sensory assessment of color intensity was consistent with an earlier colorimetric analysis. Fragrance of all the samples were evaluated as little intense or unremarkable and rated at similar levels of intensity (29–34). No assessments of this parameter were found to deviate. In general, the assessors described the emulsions based on non-interesterified fats as greasier and tightly covering the skin. E9, with a 1:1 ratio of animal to vegetable fats, was evaluated as the one with the highest greasiness.

### 3.6. Consumer Testing

**Figure 7:**
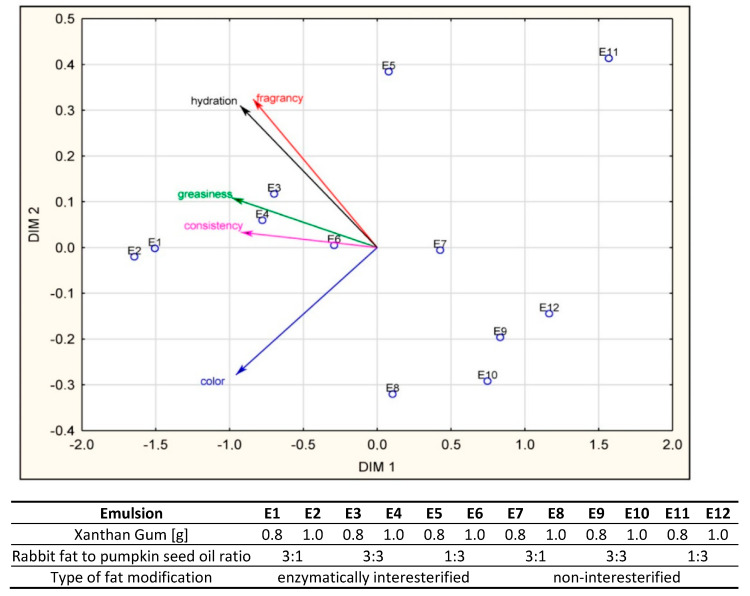
Emulsion perception map (E1–E12) in a system of variables sensory characteristics in the opinion of consumers.

Figure 7 presents a perception map derived from results of consumer assessments. The value of the STRESS factor for multidimensional scaling including all the characteristics was 0.014, indicating a good match of the matrices of reproduced distances and observed distances [30].

The results of the PROFIT analysis including coordinates of the characteristics help to evaluate similarities and differences between samples under assessment [52]. Błażejczyk-Majka and Boczar [53] stated that vector senses indicate the rising value of a characteristic a vector denotes. Thus, the vector distribution in Figure 7 shows that desirability assessments are determined by three variable groups: color, consistency and greasiness, fragrance and hydration. It should be noted that distances of objects tested from characteristic vectors are not important to the interpretation of results. However, the array of such characteristic projections on to a vector is of importance. Therefore, it can be noted that the samples E1 and E2 are not only highly similar with regard to sensory characteristics but are also best rated for all the characteristics studied.

Thus, this indicated that the most accurately selected fat composition in the presented emulsions were those that contained more rabbit fat than vegetable oil. Enzymatically modified fats utilized as emulsion fatty bases contributed to positive assessments of these emulsions by consumers. Their consistency, spreadability on the skin, skin hydration, and skin comfort after their application were more satisfactory (Figure 6). A comparison between results of the consumer assessment and profiles obtained from testing by the sensory team demonstrated that the emulsions displaying more compact consistency, less intensive color, and producing less greasy sensations tended to be more appreciated. The desirability profile of these characteristics clearly pointed to the emulsions based on modified fats. Consumer evaluations and intensity assessments by the sensory team varied somewhat with reference to odor. The odor intensity of all the emulsions was very similar in the belief of all the assessors. Consumers did find some differences in odor desirability, on the other hand. Lawless and Heymann [23] suggested that such variations may result from impact of one characteristic under assessment on others, the so-called halo effect. The photographs (Figure 5) and instrumental color measurements (Table 2) proved that the samples did differ from one another. Since color was the first characteristic to be appraised by the consumers, it could have had a direct impact on the evaluation of the subsequent feature, i.e., odor. The darker emulsions E7–E12 were rated distinctly lower in terms of odor preferences than E1-E6 despite the fact that the odor intensities of these emulsions were very similar (as rated by the sensory team). The consumers were presumably led by darker emulsion color to rate odor of the same emulsions more negatively. The supposition accords with the conclusions of [54,55] who have confirmed a change of cream color alone may affect the assessment of other sensory characteristics.

## 4. Conclusions

The study proved that the dispersion systems based on both unmodified fats and structured lipids exhibited no significant destabilization in visual assessment. On the other hand, the Turbiscan test indicated some destabilization changes for the emulsions containing modified fat with greater rabbit fat content. The results of the sensory assessment showed that the evaluating panel awarded higher scores to the emulsions based on enzymatically modified fats. They found the emulsions to display thicker and more homogeneous consistency and better spreadability on the skin. The sensory and consumer assessments indicated the emulsions containing the lowest share of pumpkin seed oil in the modified fats that constituted the fatty bases were rated the best. This may suggest the color arising from the pumpkin seed oil clearly dominated the overall evaluation of these emulsions by the sensory panel and the consumers. The physicochemical analysis and sensory assessment of the emulsion systems indicated a need to expand the research towards an improvement of the systems’ stability. Emulsions based on structured fat containing the same proportion of animal fat and pumpkin seed oil seemed like the optimum solution. The results obtained in the work also confirmed that both the amount of 0.8 and 1.0% of hydrocolloid in the presented emulsions similarly stabilized them. Therefore, from an economic point of view, it is reasonable to work on smaller quantities of this component. 

Although no close coherence between stability and consumer acceptance could be observed, the results confirmed a major role of sensory evaluation in the development of new emulsion products. They also provide knowledge on modifications to product characteristics that would lead to the best possible quality and consumer acceptance.

This research has also reaffirmed that looking for new fats among waste fats is becoming a solution to finding new fatty bases for emulsions. The natural origin of these ingredients, and thus their agreeability with the human body, appear noteworthy as well. The application of enzymatic modification of rabbit fat is a waste-free technique. Enrichment with unsaturated fatty acids is an added advantage of enzymatic modification of rabbit fat with pumpkin seed oil and can be applied not only for food but also for skin applications.

## Figures and Tables

**Table 1:** Composition and type of produced emulsions.

Emulsions	Fat Type					Component (%) wt/wt	
				Xanthan Gum [g]	Emulsifiers * [%]		Water
					MAG	DAG	
E1	RF:PSO	3:1	EIE blends	0.8			Up to 100.0
E2				1.0			
E3		3:3		0.8			
E4				1.0	4.8	21.00	
E5		1:3		0.8			
E6				1.0			
E7	RF:PSO	3:1	NIE blends	0.8			
E8				1.0			
E9		3:3		0.8			
E10				1.0	-	-	
E11		1:3		0.8			
E12				1.0			

RF—rabbit fat; PSO—pumpkin seed oil; EIE—enzymatically interesterified; NIE—non-interesterified; MAG—monoacylglycerols, DAG—diacylglycerols; * products formed during enzymatic modification.

**Table 2:** Changes in color parameters of the prepared emulsions after 24 h and 30 days from their preparation (mean values ± standard deviation).

Emulsions	24 h				30 Days				
	L*	a*	b*	C*	L*	a*	b*	C*	∆E
E1	18.0 ± 0.8	0.6 ± 0.4	1.1 ± 0.3	1.2 ± 0.5	15.0 ± 0.5	−0.3 ± 0.1	1.9 ± 0.2	1.8 ± 0.3	2.7 ± 0.1
E2	17.9 ± 0.3	0.8 ± 0.1	1.1 ± 0.2	1.3 ± 0.2	15.7 ± 0.5	−0.8 ± 0.1	3.3 ± 0.1	3.4 ± 0.2	1.9 ± 0.2
E3	17.4 ± 1.0	1.2 ± 0.1	0.7 ± 0.5	1.4 ± 0.5	13.8 ± 0.2	−0.5 ± 0.1	2.8 ± 0.1	2.9 ± 0.1	3.3 ± 0.3
E4	18.2 ± 0.7	1.1 ± 0.1	1.1 ± 0.3	1.6 ± 0.3	14.2 ± 0.2	−0.7 ± 0.1	3.0 ± 0.1	3.0 ± 0.1	3.8 ± 0.2
E5	18.2 ± 0.4	0.9 ± 0.1	0.3 ± 0.3	1.0 ± 0.2	14.2 ± 0.2	−0.6 ± 0.1	2.7 ± 0.1	2.7 ± 0.1	3.8 ± 0.1
E6	18.2 ± 0.5	0.8 ± 0.1	0.6 ± 0.6	1.0 ± 0.4	13.6 ± 0.1	−0.6 ± 0.1	2.6 ± 0.1	2.7 ± 0.1	4.4 ± 0.3
E7	17.1 ± 0.3	1.5 ± 0.1	−0.2 ± 0.1	1.5 ± 0.2	13.9 ± 0.2	−0.4 ± 0.1	2.0 ± 0.2	2.1 ± 0.1	3.1 ± 0.1
E8	17.4 ± 0.9	1.7 ± 0.1	−0.2 ± 0.1	1.7 ± 0.2	13.0 ± 0.4	−0.5 ± 0.2	1.8 ± 0.1	1.9 ± 0.2	4.4 ± 0.3
E9	16.7 ± 0.2	1.7 ± 0.1	−0.3 ± 0.2	1.7 ± 0.2	12.2 ± 0.2	−0.5 ± 0.2	1.6 ± 0.3	1.7 ± 0.2	4.5 ± 0.2
E10	17.2 ± 0.8	1.6 ± 0.1	−0.7 ± 0.2	1.8 ± 0.4	13.3 ± 0.4	−0.4 ± 0.1	1.6 ± 0.1	1.7 ± 0.2	3.9 ± 0.2
E11	15.8 ± 0.6	1.9 ± 0.2	−0.8 ± 0.1	2.1 ± 0.3	12.7 ± 0.5	−0.6 ± 0.3	1.2 ± 0.1	1.3 ± 0.2	3.2 ± 0.4
E12	16.3 ± 0.6	2.0 ± 0.1	−0.6 ± 0.3	2.1 ± 0.3	12.3 ± 0.5	−0.6 ± 0.1	1.5 ± 0.3	0.4 ± 0.3	4.0 ± 0.2

## Data Availability

Data is contained within the article.

## References

[1] Kumar S., Dhir A., Talwar S., Chakraborty D., Kaur P. (2021). What drives brand love for natural products? The moderating role of household size. J. Retail. Consum. Serv..

[2] Kahraman A., Kazancoglu I. (2019). Understanding consumers’ purchase intentions toward natural-claimed products: A qualitative research in personal care products. Bus. Strat. Environ..

[3] (2019). Statista. 2019. Global Market Value for Natural and Organic Cosmetics from 2018 to 2027. https://www.statista.com/statistics/673641/global-market-value-for-natural-cosmetics/.

[4] Dickinson E. (2015). Colloids in Food: Ingredients, Structure, and Stability. Annu. Rev. Food Sci. Technol..

[5] Chang Y., McClements D.J. (2016). Influence of emulsifier type on the in vitro digestion of fish oil-in-water emulsions in the presence of an anionic marine polysaccharide (fucoidan): Caseinate, whey protein, lecithin, or Tween 80. Food Hydrocoll..

[6] Bai L., Huan S., Li Z., McClements D.J. (2017). Comparison of emulsifying properties of food-grade polysaccharides in oil-in-water emulsions: Gum arabic, beet pectin, and corn fiber gum. Food Hydrocoll..

[7] Lam R.S., Nickerson M.T. (2013). Food proteins: A review on their emulsifying properties using a structure–function approach. Food Chem..

[8] Rosalam S., England R. (2016). Review of xanthan gum production from unmodified starches by *Xanthomonas campestris* sp.. Enzyme Microb. Technol..

[9] Desplanques S., Renou F., Grisel M., Malhiac C. (2012). Impact of chemical composition of xanthan and acacia gums on the emulsification and stability of oil-in-water emulsions. Food Hydrocoll..

[10] Hanazawa T., Murray B.S. (2014). The influence of oil droplets on the phase separation of protein–polysaccharide mixtures. Food Hydrocoll..

[11] Marzec A., Kowalska H., Suwińska S. (2017). Wpływ rodzaju i zawartości tłuszczu na właściwości akustyczne ciastek kruchych. Acta Agrophysica..

[12] Kowalska M., Woźniak M., Krzton-Maziopa A., Tavernier S., Pazdur Ł., Żbikowska A. (2018). Development of the emulsions containing modified fats formed via enzymatic interesterification catalyzed by specific lipase with various amount of water. J. Dispers. Sci. Technol..

[13] Kowalska M., Mendrycka M., Zbikowska A., Kowalska D. (2017). Assessment of a stable cosmetic preparation based on enzymatic interesterified fat, proposed in the prevention of atopic dermatitis. Acta Pol. Pharm. Drug Res..

[14] Rojas V.M., Marconi L.F.D.C.B., Guimarães-Inácio A., Leimann F.V., Tanamati A., Gozzo Ângela M., Fuchs R.H.B., Barreiro M.F., Barros L., Ferreira I.C.F.R. (2019). Formulation of mayonnaises containing PUFAs by the addition of microencapsulated chia seeds, pumpkin seeds and baru oils. Food Chem..

[15] Gutierrez R.M.P. (2016). Review of Cucurbita pepo (Pumpkin) its Phytochemistry and Pharmacology. Med. Chem..

[16] Rasinska E., Czarniecka-Skubina E., Rutkowska J. (2018). Fatty acid and lipid contents differentiation in cuts of rabbit meat. CyTA J. Food.

[17] Leiber F., Meier J.S., Burger B., Wettstein H.-R., Kreuzer M., Hatt J.-M., Clauss M. (2008). Significance of Coprophagy for the Fatty Acid Profile in Body Tissues of Rabbits Fed Different Diets. Lipids.

[18] Givens D.I. (2009). Animal nutrition and lipids in animal products and their contribution to human intake and health. Nutrients.

[19] Zotte A.D., Szendrő Z. (2011). The role of rabbit meat as functional food. Meat Sci..

[20] Stokman H., Gevers T., Koenderink J. (2000). Color Measurement by Imaging Spectrometry. Comput. Vis. Image Underst..

[21] Klimaszewska E., Seweryn A., Małysa A., Zieba M., Lipińska J. (2018). The effect of chamomile extract obtained in supercritical carbon dioxide conditions on physicochemical and usable properties of pharmaceutical ointments. Pharm. Dev. Technol..

[22] Zhu Q., Wu F., Saito M., Tatsumi E., Yin L. (2016). Effect of magnesium salt concentration in water-in-oil emulsions on the physical properties and microstructure of tofu. Food Chem..

[23] Lawless H.T., Heymann H. (2010). Sensory Evaluation of Food. Principles and Practices.

[24] ISO 4121. Sensory Analysis - Guidelines for the Use of Quantitative Response Scales. Genewa: ISO. 2003, 1–9. https://www.iso.org/standard/33817.html.

[25] ISO 11132. Sensory Analysis - Methodology - Guidelines for Monitoring the Performance of a Quantitative Sensory Panel. Genewa: ISO. 2012, 1–23. https://www.iso.org/standard/50124.html.

[26] ISO 5496. Sensory Analysis - Methodology - Initiation and Training of Assessors in the Detection and Recognition of Odours. Genewa: ISO. 2006, 1–16. https://www.iso.org/standard/44247.html.

[27] ISO 6658. Sensory Analysis - Methodology - General Guidance. Genewa: ISO. 2017, 1–26. https://www.iso.org/standard/65519.html.

[28] ISO 8586. Sensory Analysis - General Guidelines for the Selection, Training and Monitoring of Selected Assessors and Expert Sensory Assessors. Genewa: ISO. 2012, 1–26. https://www.iso.org/standard/45352.html.

[29] ISO 11136. Sensory analysis - Methodology - General guidance for conducting hedonic tests with consumers in a controlled area. Genewa: ISO. 2014, 1-44. https://www.iso.org/standard/50125.html.

[30] Zaborski A. (2009). Skalowanie Wielowymiarowe. Statystyczna Analiza Danych z Wykorzystaniem Programu R.

[31] Quan T.H., Benjakul S., Quân T.H. (2018). Gelling properties of duck albumen powder as affected by desugarization and drying conditions. J. Texture Stud..

[32] Chandra M.V., Shamasundar B. (2015). Texture Profile Analysis and Functional Properties of Gelatin from the Skin of Three Species of Fresh Water Fish. Int. J. Food Prop..

[33] Assadpour E., Maghsoudlou Y., Jafari S.M., Ghorbani M., Aalami M. (2016). Optimization of folic acid nano-emulsification and encapsulation by maltodextrin-whey protein double emulsions. Int. J. Biol. Macromol..

[34] Kowalska M., Babut M., Woźniak M., Żbikowska A. (2019). Formulation of oil-in-water emulsions containing enzymatically modified rabbit fat with pumpkin seed oil. J. Food Process. Preserv..

[35] Papalamprou E.M., A Makri E., Kiosseoglou V.D., I Doxastakis G. (2005). Effect of medium molecular weight xanthan gum in rheology and stability of oil-in-water emulsion stabilized with legume proteins. J. Sci. Food Agric..

[36] Saharudin S.H., Ahmad Z., Basri M. (2016). Role of xanthan gum on physicochemical and rheological properties of rice bran oil emulsion. Int. Food Res. J..

[37] McClements D.J. (2005). Food Emulsions: Principles, Practices, and Techniques.

[38] Chudy S., Gierałtowska U., Krzywdzińska-Bartkowiak M., Piątek M. (2016). Pomiar barwy produktów mleczarskich. Współczesne Trendy w Kształtowaniu Jakości żywności.

[39] Weiss J., McClements D.J. (2001). Color changes in hydrocarbon oil-in-water emulsions caused by Ostwald ripening. J. Agric. Food Chem..

[40] Chantrapornchai W., Clydesdale F., McClements D.J. (2001). Influence of Flocculation on Optical Properties of Emulsions. J. Food Sci..

[41] Liu Z.Q., Yang X., Zhang Q. (2014). TURBISCAN: History, Development, Application to Colloids and Dispersions. Adv. Mater. Res..

[42] Liu J., Huang X.-F., Lu L., Li M.-X., Xu J.-C., Deng H.-P. (2011). Turbiscan Lab^®^ Expert analysis of the biological demulsification of a water-in-oil emulsion by two biodemulsifiers. J. Hazard. Mater..

[43] LeMarchand C. (2003). Study of emulsion stabilization by graft copolymers using the optical analyzer Turbiscan. Int. J. Pharm..

[44] McClements D.J., Rao J. (2011). Food-Grade Nanoemulsions: Formulation, Fabrication, Properties, Performance, Biological Fate, and Potential Toxicity. Crit. Rev. Food Sci. Nutr..

[45] Gao Z., Fang Y., Cao Y., Liao H., Nishinari K., Phillips G.O. (2017). Hydrocolloid-food component interactions. Food Hydrocoll..

[46] Raymundo A., Franco J.M., Empis J., De Sousa I.M.N. (2002). Optimization of the composition of low-fat oil-in-water emulsions stabilized by white lupin protein. J. Am. Oil Chem. Soc..

[47] Vélez G., Fernández M.A., Muñoz J., Williams P.A., English R.J. (2003). Role of Hydrocolloids in the Creaming of Oil in Water Emulsions. J. Agric. Food Chem..

[48] Traynor M., Burke R., Fria J.M. (2013). Gaston, E.; Barry-Ryan, C. Formation and stability of an oil in water emulsion containing lecithin, xanthan gum and sunflower oil. Int. Food Res. J..

[49] Xu D., Zhang J., Cao Y., Wang J., Xiao J. (2016). Influence of microcrystalline cellulose on the microrheological property and freeze-thaw stability of soybean protein hydrolysate stabilized curcumin emulsion. LWT.

[50] Ren Y., Zheng J., Xu Z., Zhang Y., Zheng J. (2018). Application of Turbiscan LAB to study the influence of lignite on the static stability of PCLWS. Fuel.

[51] Moravkova T., Filip P. (2016). Relation between sensory analysis and rheology of body lotions. Int. J. Cosmet. Sci..

[52] Jabkowski P. (2010). O korzyściach wynikających z zastosowania analizy PROFIT. Praktyczna Analiza Danych w Marketingu i Bada-Niach Rynku.

[53] Błażejczyk-Majka L., Boczar P. (2016). Zastosowanie metod wielowymiarowych w charakterystyce preferencji konsumentów. Metody Ilościowe w Badaniach Ekonomicznych.

[54] Turek P., Głowik A. (2015). The Effects of the Moisturizing Cream Color on the Perception of Selected Sensory Attributes. Current Trends in Commodity Science: Development and Assessment of Non-food Products.

[55] Turek P. (2017). Color Modification of the Face Cream and its General Sensory Quality. Polish J. Commod. Sci..

